# Religious and Spiritual Factors in Depression

**DOI:** 10.1155/2012/298056

**Published:** 2012-09-18

**Authors:** Sasan Vasegh, David H. Rosmarin, Harold G. Koenig, Rachel E. Dew, Raphael M. Bonelli

**Affiliations:** ^1^Department of Psychiatry, Ilam University of Medical Sciences, Ilam, Iran; ^2^Department of Psychiatry, Harvard Medical School, Boston, MA 02215, USA; ^3^Center for Spirituality, Theology and Health, Duke University Medical Center, Durham, NC 27705, USA; ^4^Department of Medicine, King Abdulaziz University, Jeddah, 21589, Saudi Arabia; ^5^Division of Adolescent Psychiatry, Duke University Medical Center, Durham, NC 27710, USA; ^6^Departments of Psychiatry and Neurology, Sigmund Freud University, Vienna, 1030, Austria

To our knowledge, this issue is the first peer-reviewed journal in psychiatry to devote an entire issue to this subject and is certainly the first open access journal to do so. For nearly a century, mental health professionals have been reluctant to conduct systematic research on religion and depression—almost as reluctant as they have been to address it in therapy. Freud viewed religion as a form of neurosis [1–3], and other mental health professionals over the years have likewise argued that the less religious that people are, the healthier they will be [4–6]. 

As a result, when religion came up in the clinical counter, it was either ignored or treated as part of the pathology that had to be corrected in the treatment [7]. By the end of the 20th century, this negative attitude towards religion had impacted the personal views of many psychologists and psychiatrists themselves. Surveys during this period found that from 57% to 74% of psychologists [8, 9] and from 24% to 75% of psychiatrists [10–12] did not believe in God, compared to only 4% in the general US population [13]. This pathological view of religion had even become institutionalized as part of the psychiatric nomenclature. A systematic review of the religious content of DSM-III-R found that over 22% of all cases of mental illness included religious descriptions [14]. 

There is indication in recent years, however, that attitudes toward religion in psychiatry are changing. Consider a recent national survey of U.S. psychiatrists that found 84% of psychiatrists indicating a religious affiliation, 74% describing themselves as moderately or highly spiritual, 65% believing in God, and 29% attending religious services at least twice a month or more [15, 16]. Furthermore, 76% said that the influence of religion/spirituality on health is generally positive, and 64% that religion/spirituality often or always “gives patients a positive, hopeful state of mind [17].” Likewise, a national survey of US psychologists around the same time reported similar findings; 82% regarded religion as beneficial rather than harmful to mental health [18]. One reason for this change in attitude is the outpouring of systematic research on religion and mental health. Over the past 30 years, the generally negative view in psychiatry toward religion (based primarily on the opinion of influential mental health professionals quoted above) has been challenged by hundreds of observational studies and a handful of randomized clinical trials—nearly 300 in depression alone [19]. 

Adding to the research base on religion and depression are the studies reported in this special issue. The lead article (authored by the editors) is a review up through 2010 of quantitative research on religion, spirituality and depression, including both observational studies and randomized clinical trials, and brief mention of more recent studies published earlier this year. Research findings, both the positive and negative, are reviewed, and reasons for them are discussed. The article ends by an examination of religious psychotherapies that illustrate ways of integrating patients' religious beliefs into therapy. This review sets the tone for the original research reports that follow.

The research published in this issue by T. Sorensen and colleagues from, Norway, analyzes data from the HUNT-3 study (a population-based study of 37,981 participants) in order to examine how religious involvement (religious attendance) moderates the relationship between death of a family member and depressive symptoms. This is one of the few studies from secular Northern Europe that examines these relationships. The population-based aspect of this study is particularly important in generalizing the findings.

In another research report, also from Europe (the Netherlands), A. W. Braam and colleagues examine the relationship between religious involvement and depressive symptoms among those at the end of life. Data come from the Amsterdam Longitudinal Study of Aging that include 272 after-death proxy interviews with family members of deceased participants. Relationships between religious involvement, feelings of depression, and feelings of peace during the last week of life were analyzed, controlling for a wide range of other predictors. 

In a report by L. L. Toussaint and colleagues from Luther College and Harvard University, researchers examine the possible mediating role that forgiveness plays in the relationship between religiousness/spirituality and depression. This prospective study involves a nationally representative sample of 966 U.S. adults assessed at baseline and six months later. Religious involvement was measured at baseline by attendance at services, frequency of prayer, and self-ratings of spirituality and religion. Four dimensions of forgiveness were assessed (forgiving self, God, others, and seeking forgiveness). Depressive disorder was determined using the World Health Organization's Composite International Diagnostic Interview (CIDI) both at baseline and follow-up.

An article by L. L. Hourani and colleagues assesses the influence of spirituality on depression, posttraumatic stress disorder (PTSD), and suicidal tendencies in 24,000 randomly selected activity duty military personnel. Data were analyzed from a 2008 U.S. Department of Defense survey examining health-related behaviors of soldiers in the army, navy, air force, and marine corp. Spirituality was assessed by agreement with the statements, “My religious/spiritual beliefs are a very important part of my life” and “My religious/spiritual beliefs influence how I make decision in my life.” Depression was measured with the 10-item Center for Epidemiological Studies-Depression scale, PTSD was assessed with a 17-item self-report measure, and suicidal tendencies were examined with two items asking about suicidal thoughts and suicidal attempts within the past year.

Next, R. D. Hayward and colleagues at Duke University use structural equation modeling to examine the longitudinal relationship between religious involvement (subjective religiosity, private prayer, attendance at services, and religious media use) and clinician-rated depression severity using the Montgomery-Asberg Depression Rating Scale (MADRS). Participants were ages 59 or over and diagnosed with unipolar depression at the baseline interview (when religious characteristics were also measured). The MADRS was used to assess depressive symptoms at baseline and three months later. Both direct and indirect effects of religious variables were examined on change in depressive symptoms, controlling for other participant characteristics.

Acquiring a mixture of qualitative and quantitative data, L. Blalock and R. E. Dew survey 25 clergy regarding mental health issues in children. Examined were clergy referral habits, knowledge about childhood mental disorders, past experiences with mental health provides, and resources available in their local religious communities. Clergy came from a variety of religious traditions, including Protestant Christian, Jewish, Hindu, and Muslim. The findings provide important information on how members of the clergy in central North Carolina (part of the Bible Belt) behave and feel toward mental health professionals who provide care to children in their congregations.

John Peteet from the Dana Farber Cancer Institute (part of Harvard Medical School) begins to address how religion/spirituality can be incorporated into clinical practice. Although no original data is included in this report, he provides a conceptual framework for integrating spirituality into the treatment of depression. He discusses the obstacles that impede such integration, presents an approach to those obstacles, and provides a rationale for including spirituality in assessment, formulation, and treatment.

Finally, an article by H. G. Koenig describes an ongoing randomized clinical trial of religious versus conventional cognitive-behavioral therapy (CBT) for major depression in patients with chronic medical illness. The rationale behind the study, the specific aims, methodology, and preliminary results are described. The therapy is being provided remotely by either instant messaging, Skype, or by telephone. Religious CBT is being conducted using a manualized intervention adapted to the specific religious traditions of participants in the study (Christian, Buddhist, Hindu, Jewish, and Muslim). Outcomes being examined include depressive symptoms (Beck), positive behaviors/emotions (gratefulness, optimism, and generosity), and indicators of immune/endocrine function. Also being examined is the influence of genetic polymorphisms on treatment response. This study represents a prototype of the kinds of studies that are needed to help guide the use of religion/spirituality in the treatment of depression.

In summary, studies in this special issue involve a wide range of research designs from cross-sectional studies to prospective studies to randomized clinical trials. Both the theory behind using religious/spiritual factors in the treatment of depression and the barriers to doing so are discussed. We hope that the research and discussions presented in this issue will help to focus attention on what may be an important resource (and sometimes liability) for depressed persons, one that is often neglected by mental health professionals. It is a resource that the majority of populations around the world and throughout recorded history have told us helps them to deal with the difficult situations and circumstances that often lie at the root of depression. May we now begin to listen to them.

*Sasan Vasegh* *Sasan Vasegh* *David H. Rosmarin* *David H. Rosmarin* *Harold G. Koenig* *Harold G. Koenig* *Rachel E. Dew* *Rachel E. Dew* *Raphael M. Bonelli* *Raphael M. Bonelli*
